# Cellular Epigenetic Targets and Epidrugs in Breast Cancer Therapy: Mechanisms, Challenges, and Future Perspectives

**DOI:** 10.3390/ph18020207

**Published:** 2025-02-03

**Authors:** Ibrahim S. Alalhareth, Saleh M. Alyami, Ali H. Alshareef, Ahmed O. Ajeibi, Manea F. Al Munjem, Ahmad A. Elfifi, Meshal M. Alsharif, Seham A. Alzahrani, Mohammed A. Alqaad, Marwa B. Bakir, Basel A. Abdel-Wahab

**Affiliations:** 1 College of Pharmacy, Najran University, Najran 66256, Saudi Arabia; ibra11him1999@gmail.com (I.S.A.); salehalharth@hotmail.com (S.M.A.); 2 Department of Pharmaceuticals Care, Ministry of Defense, Najran 66281, Saudi Arabia; ph_771@hotmail.com (A.H.A.); ahmad.ajeebi2277@gmail.com (A.O.A.); ahmad.elfifi@gmail.com (A.A.E.); meshal.m.990@gmail.com (M.M.A.); 3 King Khaled Hospital -Najran Health Cluster, Najran 66261, Saudi Arabia; maneafaresalm33@hotmail.com; 4 Pharmacy Department, Khamis Mushait General Hospital, King Khalid Rd, Al Shifa, Khamis Mushait 62433, Saudi Arabia; sealzhrani@moh.gov.sa; 5 Department of Pharmaceutical Care Services, Al Noor Specialized Hospital, Makkah Health, Cluster, Makkah 24241, Saudi Arabia; malqaad@moh.gov.sa; 6 Department of Medical Education, College of Medicine, Najran University, Najran 1988, Saudi Arabia; mbbakir@nu.edu.sa; 7 Department of Pharmacology, College of Pharmacy, Najran University, Najran 1988, Saudi Arabia

**Keywords:** Breast cancer, epigenetics, epidrugs, DNA methylation, histone modification, histone acetyl-transferase and histone deacetylase inhibitors, histone methyltransferase and demethyltransferase inhibitors

## Abstract

Breast cancer is the most common malignancy affecting women, manifesting as a heterogeneous disease with diverse molecular characteristics and clinical presentations. Recent studies have elucidated the role of epigenetic modifications in the pathogenesis of breast cancer, including drug resistance and efflux characteristics, offering potential new diagnostic and prognostic markers, treatment efficacy predictors, and therapeutic agents. Key modifications include DNA cytosine methylation and the covalent modification of histone proteins. Unlike genetic mutations, reprogramming the epigenetic landscape of the cancer epigenome is a promising targeted therapy for the treatment and reversal of drug resistance. Epidrugs, which target DNA methylation and histone modifications, can provide novel options for the treatment of breast cancer by reversing the acquired resistance to treatment. Currently, the most promising approach involves combination therapies consisting of epidrugs with immune checkpoint inhibitors. This review examines the aberrant epigenetic regulation of breast cancer initiation and progression, focusing on modifications related to estrogen signaling, drug resistance, cancer progression, and the epithelial–mesenchymal transition (EMT). It examines existing epigenetic drugs for treating breast cancer, including agents that modify DNA, inhibitors of histone acetyltransferases, histone deacetylases, histone methyltransferases, and histone demethyltransferases. It also delves into ongoing studies on combining epidrugs with other therapies and addresses the upcoming obstacles in this field.

## 1. Introduction

**Figure 1:**
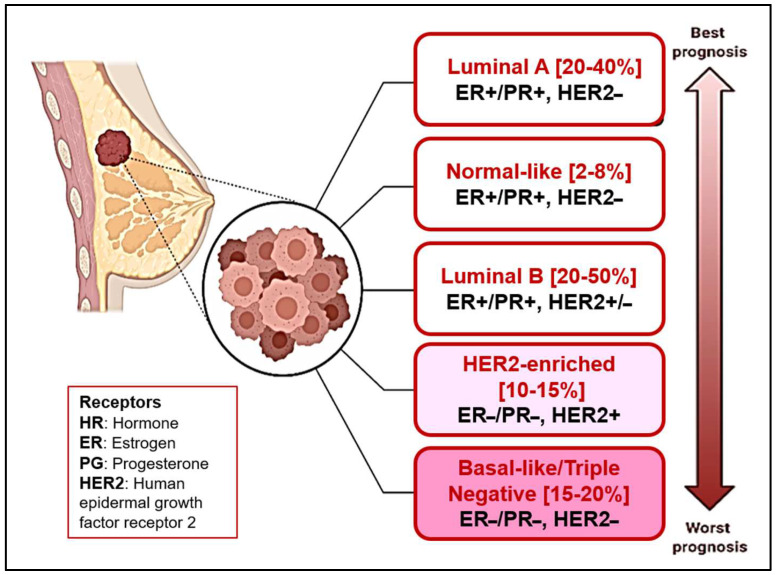
Intrinsic or molecular subtypes of breast cancer (BC).

Breast cancer is categorized into five molecular classes: (1) luminal A, (2) luminal B, (3) HER2-overexpressing, (4) normal, and (5) triple-negative breast cancer (TNBC) (Figure 1) [1].

The hormone-receptor-positive (HR+) subtype encompasses cancer cells that express the estrogen receptor (ER) and progesterone receptor (PR), which stimulate cancer cell growth. Hormonal therapy targeting these receptors is frequently an efficacious treatment approach for this class [2]. The class of HER2-positive BC cells is characterized by an overexpression of the HER2 receptor, which results in increased aggression [3]. One more significant class, TNBC, is clearly identified by the absence of both estrogen, progesterone, and HER2/neu receptors. This lack of hormonal receptors renders TNBC unresponsive to conservative hormone-based therapies and targeted medications that specifically address these receptors [4].

TNBC’s heterogeneous nature presents a significant therapeutic challenge. TNBC is clinically identified by a lack of hormone receptors and an absence of human epidermal growth factor receptor 2 (HER2) [5]. Pathologically, TNBC exhibits aggressive and invasive characteristics, including high rates of cell division, growth, and metastasis of malignant cells, resulting in fast disease development and poor outcomes [6]. TNBC exhibits heterogeneity and genetic alterations at the levels of both genes and cells, as evidenced by the prevalence of serious mutations and varied immune profiles within TNBC tissues [7,8].

The clinical management of TNBC is significantly impeded by the absence of crucial molecular targets, including hormone receptors and HER2. A multifaceted approach combining hormonal, surgical, radio, and targeted therapies is usually employed to address TNBC [9]. Currently, the primary non-surgical approach for treating systemic triple-negative breast cancer (TNBC) relies on cytotoxic chemotherapy agents [10]. Recent advancements in triple-negative breast cancer (TNBC) chemotherapy have facilitated more personalized approaches, wherein drug selection and treatment regimens are tailored to address the specific requirements of individual patients [11]. However, complete response is achieved by only a small proportion of TNBC patients, specifically less than 30% [12]. Moreover, the efficacy of conventional chemotherapeutic agents is impeded by the frequent occurrence of chemotherapy-induced recurrence in TNBC patients undergoing treatment, particularly in advanced stages of TNBC [13,14].

Novel strategies for the treatment of TNBC have emerged, including immunotherapeutic approaches such as the use of immune checkpoint blockers (ICBs) and chimeric antigen receptor T cell (CAR-T) therapies [15,16]. In comparison to the other types of breast cancer subtypes, TNBC exhibits a higher ability of infiltration of the immune cells and an elevated tendency for immune checkpoint molecule expression, including cytotoxic T-lymphocyte-associated protein 4 (CTLA-4) and programmed death-ligand 1 (PD-L1) [17]. This distinctive characteristic renders TNBC a more suitable candidate for immunotherapeutic interventions, including CAR-T-directed cell therapies and PD-L1-specific inhibitors. At present, two monoclonal-antibody-specific inhibitors targeting the vital PD-1/PD-L1 pathway have received regulatory approval for the treatment of TNBC, while recently developed aptamers designed to disrupt PD-1/PD-L1 signaling are currently still under research [18,19]. Advanced TNBC treatment protocols typically incorporate synergistic chemotherapy drug combinations to enhance the limited efficacy of antibody monotherapy. However, the inadequate response rates of current immunotherapies underscore the necessity for ongoing research and innovation in TNBC treatment approaches. Despite notable advancements in treatment efficacy over time, several obstacles have emerged, including severe adverse effects, multidrug resistance development, intratumoral heterogeneity, and metastatic disease [13]. Beyond the extensively researched genetic alterations, epigenetic modifications contribute significantly to breast cancer development by promoting aberrant gene expression. The growing understanding of cancer epigenetics offers novel therapeutic avenues for breast cancer treatment [20]. While genetic alterations, including the induction of oncogenes or impairment of tumor suppressor genes, contribute to tumor progression and the outcomes of breast cancer treatment, these genetic factors alone do not provide a comprehensive explanation for the behavior of cancer cells and their complex interactions with the surrounding tumor microenvironment (TME) through the treatment journey [21]. Epigenetic dysregulation has been associated with TNBC heterogeneity, treatment resistance, and malignant progression [22]. Notably, the patterns of methylation of DNA and modifications of histone are differentially regulated across TNBC subtypes. Altering such modifications can impact antitumor immune responses and anticancer drug effectiveness in a mouse model of mesenchymal TNBC [23]. Consequently, modifying TNBC epigenetic patterns presents a promising strategy for enhancing immunotherapy outcomes. Each subtype of breast cancer exhibits unique genetic and epigenetic profiles that influence tumor behavior and response to therapy. While TNBC has been a primary focus of epigenetic research due to its aggressive phenotype and limited treatment options, emerging evidence highlights the importance of epigenetic dysregulation across all breast cancer subtypes. For instance, luminal subtypes often exhibit epigenetic alterations in estrogen receptor signaling pathways, while HER2-enriched tumors may involve the epigenetic regulation of HER2 amplification [24]. The efficacious use of epidrugs in hematological cancers has sparked curiosity about their possible use in solid tumor treatment [25]. Preliminary research suggests that these pharmacological agents may augment the efficacy of conventional therapies on treatment-resistant neoplastic cells and specifically target cancer stem cells (CSCs), which are implicated in the initiation, development, and metastasis of drug-resistant cancer cells [26,27]. This review provides a comprehensive overview of epigenetic modifications and epigenetic targets in breast cancer development and progression, emphasizing how epigenetic dysregulation affects breast cancer cell development. Additionally, it highlights current successes and probable future applications of epimedicine, including different inhibitors and its use in therapeutic strategies of breast cancer, to offer a holistic perspective on the role of epigenetics in breast cancer pathogenesis and treatment.

## 2. Materials and Methods

This review article was conducted following a comprehensive and systematic search of the literature to identify relevant studies on epigenetic targets and epigenetic therapies in breast cancer.

### 2.1. Search Strategy

The search strategy was designed to ensure the inclusion of a broad range of studies, including preclinical and clinical research, published between January 2000 and October 2024. The following electronic databases were searched: PubMed, Scopus, Web of Science, and Google Scholar. The search terms and keywords used included breast cancer, epigenetic therapy, epidrugs, DNA methyltransferase inhibitors, histone deacetylase inhibitors, histone acetyltransferase inhibitors, histone demethylase inhibitors, and histone methyltransferase inhibitors. Boolean operators (AND, OR) were used to combine keywords and refine the search results. Additionally, reference lists of relevant articles were manually screened to identify additional studies that may not have been captured in the initial database searches.

### 2.2. Inclusion and Exclusion Criteria

Studies were included if they focused on epigenetic therapies or epidrugs in the context of breast cancer; were published in English; were original research articles, reviews, or meta-analyses; and were published between January 2000 and March 2024. Studies were excluded if they were unrelated to breast cancer or epigenetic mechanisms; published in languages other than English; or editorials, commentaries, or conference abstracts without full-text availability.

### 2.3. Data Extraction and Synthesis

Data from selected studies were extracted and organized into thematic categories, including mechanisms of action, preclinical findings, clinical trial outcomes, and challenges in the development of epigenetic therapies. The findings were synthesized to provide a comprehensive overview of the current state of research on epigenetic therapies in breast cancer.

### 2.4. Quality Assessment

The quality of included studies was assessed using established criteria for preclinical and clinical research, such as study design, sample size, reproducibility, and statistical rigor. This ensured the inclusion of high-quality evidence in the review. This systematic approach allowed for a thorough and unbiased evaluation of the literature, providing a robust foundation for the insights and conclusions presented in this review.

## 3. Breast Cancer and Epigenetic Regulation

**Figure 2:**
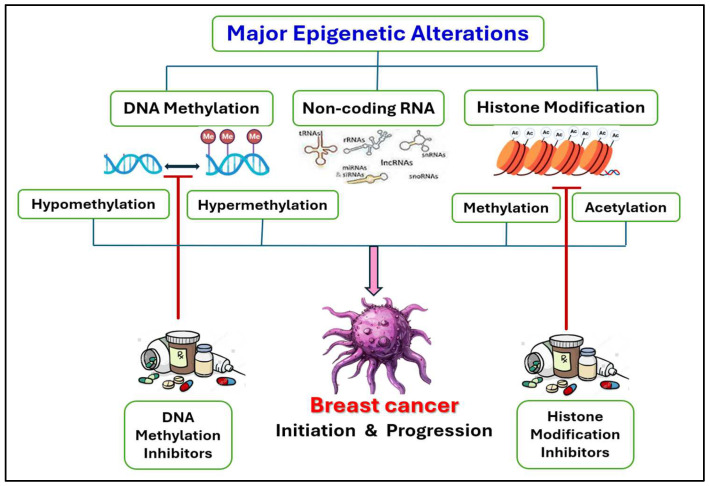
Significant epigenetic alterations of breast cancer and epidrug targets.

The development and progression of cancer have been associated with epigenetic alterations for numerous years [28,29,30]. Studies profiling tumors have revealed significant epigenomic differences between normal and cancerous samples, with tumor samples exhibiting considerable heterogeneity [31]. Moreover, epigenetic markers have demonstrated potential in breast cancer risk assessment [32], detection [33], outcome prediction [20], and treatment response evaluation [24]. Given that epigenetic alterations in cancer cells can impact on the expression of genes responsible for cell motility, invasion, and angiogenesis [34], it is rational to investigate agents that are able to modify these alterations. These epigenetic alterations also persist beyond typical gene expression patterns [35]. In addition to genetic mutations, various epigenetic modifications contribute to breast cancer initiation and progression. Heritable modifications of deoxyribonucleic acid (DNA) that influence gene expression without altering the DNA sequence are called epigenetic changes. These modifications encompass DNA methylation, alterations to histone tails, the reorganization of nucleosomes, and regulatory activities of non-coding RNA. These processes are essential for controlling the transcription of genes, maintaining genomic stability, and for sustaining development as well as the differentiation of cancer cells [36] (Figure 2).

This figure summarizes the primary epigenetic alterations associated with breast cancer, including DNA methylation, histone modifications, and non-coding RNA regulation. DNA methylation is depicted as a key mechanism involving both hypermethylation and hypomethylation, which can influence gene expression and contribute to cancer progression. Histone modifications, such as methylation and acetylation, are shown to play critical roles in regulating chromatin structure and gene activity. The figure also highlights the impact of these epigenetic changes on breast cancer development and progression, as well as the potential therapeutic targeting of these alterations using epigenetic inhibitors.

These alterations are hypothesized to contribute to the initial phases of breast cancer development and frequently represent indicators for initial detection, prognosis evaluation, and treatment efficacy. Furthermore, a comprehensive understanding of epigenetic modifications of the breast cancer cells represents a promising field for pharmaceutical development [36]. Aberrant DNA methylation patterns involve the chemical binding of a methyl group with the carbon atom in the fifth position of cytosine in the CpG dinucleotides, which constitutes a prevalent epigenetic modification. This process is dynamically regulated by DNA methyltransferases and demethylases [37]. In normal tissues, CpG nucleotides near gene promoters typically remain unmethylated, but CpG dinucleotides remote to these regions are generally methylated. This methylation pattern is critical for controlling gene expression transcription to preserve the general stability of genomic structure in physiological conditions [38]. However, cancer cells exhibit a distinct DNA methylation profile, depicted by global hypomethylation across the genome, coupled with localized hypermethylation at CpG islands [39]. Research has revealed that the DNA methylation pattern within cancer cells and its surrounding tumor microenvironment (TME) contributes to tumor development and the nature of the immune reaction with the breast cancer, thereby affecting patient outcomes [40].

## 4. Epigenetic Alterations in Breast Cancer

### 4.1. DNA Methylation in Neoplastic Cells

The dysregulation of tumor suppressor and oncogene expression represents the initial steps in breast cancer development. For example, the transcriptional silencing of crucial genes—like TMS1, BRCA1, GATA3, FOXA1, and E-cadherin—through promoter hypermethylation contributes to breast cancer initiation and progression [41]. Clinically, BRCA1 methylation negatively associates with the cure and general survival in breast cancer patients, indicating its probable importance as a prognostic indicator in breast cancer [42]. DNA methylation plays a crucial role in preserving chromosomal stability, transposable element suppression, genomic imprinting, X chromosome inactivation, and aging. It also influences certain pathologies, particularly cancer and autoimmune disorders [43]. Breast cancer cells frequently exhibit abnormal methylation patterns. Hypermethylation in the promoter region inactivates tumor suppressor genes, such as BRCA1 and CTNNB1. Conversely, breast cancer displays global DNA hypomethylation, with less 5-methylcytosine than their equivalents in normal tissue [44]. The hypomethylation of repetitive sequences of DNA is significant in cancer cells, while these regions typically remain exceedingly methylated in normal noncancerous breast cells. Furthermore, earlier studies have shown specific gene hypomethylation in breast cancer, such as γ-synuclein gene (SNCG) and hypomethylation gene 1 (MDR1) [36]. This hypomethylation correlates with the upregulated production of ADAM12 and TSPAN9 proteins in cells of breast cancer [45]. Furthermore, the ST8SIA1 gene, which encodes ganglioside D3 synthase (GD3s), exhibits elevated expression in patients with TNBC. This increased expression is accompanied by hypomethylation of the promoter region of genes and has been noticed in breast cancer cells both in vivo and in vitro [23]. The utilization of 5-azacytidine, an inhibitor of DNA methyltransferase (DNMT), provides additional evidence for the role of abnormal hypomethylation in enhancing GD3 expression in laboratory experiments [23]. Elevated GD3 expression is accompanied with poor overall survival in breast cancer patients [46]. Moreover, breast cancer patients with chemoresistant TNBC exhibit increased GD3 expression [47]. Inhibiting GD3 through shRNA or triptolide significantly reduces the production of ganglioside GD2, a biomarker of stemness of breast cancer cells, diminishing malignancy and proliferation of breast cancer cells [48]. This suppression also mitigates drug resistance in breast cancer cells through the modulation of the FAK/Akt/mTOR and Wnt/β-catenin signaling cascades [49].

### 4.2. DNA Methylation Pattern of the Breast Cancer Microenvironment

**Figure 3:**
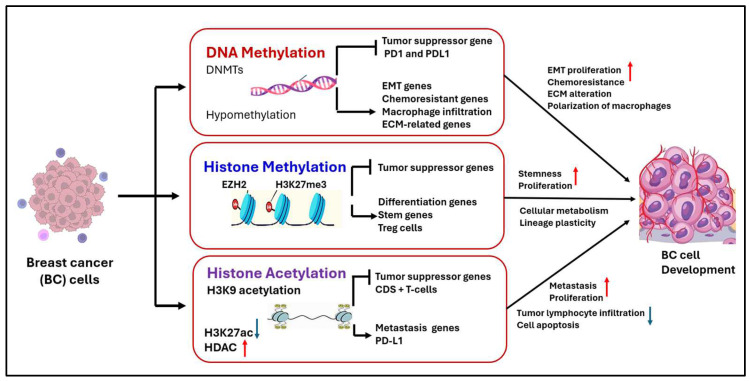
Effects of epigenetic modifications on tumor microenvironment in breast cancer. Red arrows indicate an increase, and blue arrows indicate a decrease in the mentioned parameters.

The tumor microenvironment (TME) comprises various components, including immune cells (e.g., macrophages, dendritic cells, and T lymphocytes), blood vessels, stromal cells, and matrix outside the cells [50]. The interaction between the TME and cancer cells is crucial for tumor progression and response to treatment. Aberrant DNA methylation inside the TME impacts immune responses and anti-tumor effectiveness via diverse mechanisms (Figure 3). For instance, CD8+ T cells that have been sensitized to tumors exhibit distinct DNA methylation patterns relative to normal and exhausted T cells [51]. Furthermore, methylation mediated by DNMT3B can influence macrophage polarization, and its deactivation enhances macrophage phenotypes, which are characterized by reduced inflammation [52]. Epigenetic factors also regulate the production of immunoregulatory mediators such as LAG3, CTLA4, and PD-1 in the tumor microenvironment [53] (Figure 3).

This figure illustrates the key epigenetic mechanisms—DNA methylation, histone methylation, and histone acetylation—and their roles in regulating gene expression and cellular processes in breast cancer (BC). DNA methylation is shown to influence tumor suppressor genes, immune checkpoint molecules (e.g., PD1 and PDL1), and genes involved in proliferation and differentiation. Histone methylation and acetylation are depicted as regulators of tumor suppressor genes, differentiation genes, and metastasis-related genes, impacting tumor plasticity, immune cell infiltration (e.g., macrophages and CD8+ T cells), and apoptosis. The interplay of these epigenetic modifications contributes to the progression, immune evasion, and therapeutic resistance of breast cancer cells. Abbreviations: ECN, extracellular matrix; H3K27ac, histone H3 lysine 27 acetylation; PD-L1, programmed death-ligand 1.

### 4.3. The Role of Non-Coding RNA

Non-coding RNAs (ncRNAs) are considered an additional epigenetic mechanism, accounting for approximately 62–75% of the genome [54,55]. Numerous types of ncRNAs have been identified, including circular RNAs (circRNAs), short hairpin RNAs (shRNAs), small nuclear RNAs (snoRNAs), protein-interacting RNAs (piRNAs), long non-coding RNAs (lncRNAs), and small interfering RNAs (siRNAs). These ncRNAs have become valuable sources of biomarkers and targeted therapies, enhancing our understanding of their role in various diseases [56]. MicroRNAs (miRNAs), a major category of ncRNAs, have been demonstrated to be disturbed in multifactorial disorders, including breast cancer. These short, single-stranded (18–22 nucleotides) endogenous RNA molecules bind to target mRNAs to inhibit translation. Abnormal miRNA activity has been associated with the beginning and proliferation of breast cancer [57]. For instance, metastatic breast cancer has been linked to the expression of miR-155 and miR-21 [58]. Additionally, patients diagnosed with high expression levels of miR-340-3p, miR-940, and miR-1307-3p experienced reduced overall survival [36]. Moreover, miR-497 regulates breast cancer cell growth and invasion by suppressing cyclin E1 expression [59]. Long non-coding RNAs (lncRNAs), another significant class of ncRNAs (200 nucleotides—100 KB in length), may interact with proteins, RNA, and DNA to alter gene expression at different levels [60]. Cancer development and metastasis are facilitated by the disruption of lncRNA regulatory activity. For instance, the growth-arrest-specific gene 5 (GAS5) is significantly upregulated in women with breast cancer and regulates the expression of various oncogenic genes. GAS5 can promote apoptosis in breast cancer through several signaling pathways, such as cell-death-receptor-mediated signaling pathways [61]. Furthermore, it has a crucial role in regulating the NF-κB, Wnt/β-catenin, and PI3K/AKT/mTOR pathways [62].

### 4.4. Alterations in Histone Modification

Histones are essential proteins that package DNA and maintain chromatin structure. Nucleosomes are formed when histone proteins (H2A, H2B, H3, and H4) create octamers that wrap around 147 bp of DNA. The state of chromatin can be modified through post-translational modifications (PTMs) of histones, which in turn affect gene expression regulation [63]. Various modifications occur on specific amino and carboxyl terminal residues of histone tails, such as methylation, phosphorylation, acetylation, SUMOylation, ubiquitination, ADP ribosylation, carbonylation, and glycosylation. These modifications influence gene expression. For instance, lysine residue acetylation on histones is generally associated with transcriptional activation. The cellular changes mediated by histone methylation depend on the specific residue and modified location; H3K4 methylation is linked to transcriptional activation, while H3K9 methylation is associated with transcriptional repression. Specialized enzymes targeting histone N-terminal tails catalyze these modifications [64].

These alterations are facilitated by various enzymes, including HATs, HDACs, HMTs, and HDMs. Alterations in the expression of these enzymes have been implicated in the initiation and progression of cancer [65]. During breast cancer development, numerous changes have been observed, including increased expressions of HDAC6, HBO1 p300, HDAC3, HDAC2, and HDAC1 [66]. Studies have demonstrated that histone methyltransferases, particularly KMT2, contribute to breast cancer cell growth and metastasis. KMT2 enhances oncogene- and metastasis-promoting gene activity by methylating H3K4 in both enhancer and promoter regions [67]. In breast cancer, the crucial histone methyl transferase EZH2 is frequently amplified and overexpressed. EZH2 promotes the transcriptional silencing of various genes by catalyzing H3K27 methylation, thereby inducing EMT and metastasis in breast cancer [68,69]. Additionally, another significant HMT, DOT1L, is known by its ability to enhance the metastatic potential of breast cancer [70].

Breast cancer is associated with multiple histone demethylases, including KDM4A, KDM4B, and KDM4C. In ERα-positive subtypes, KDM4A and KDM4B are overexpressed, while TNBC exhibits elevated levels of KDM4C [71]. KDM4A enhances breast cancer growth and metastasis via activating the Notch1-NICD-dependent signaling pathway [72]. KDM4B controls estrogen receptor signaling; hence, its reduced expression inhibits breast cancer progression [73]. KDM4C contributes to the progression of breast cancer via its role as an HIF-1α/VEGF signaling co-activator [74].

### 4.5. Mechanism of Epigenetics Related to Estrogen

**Figure 4:**
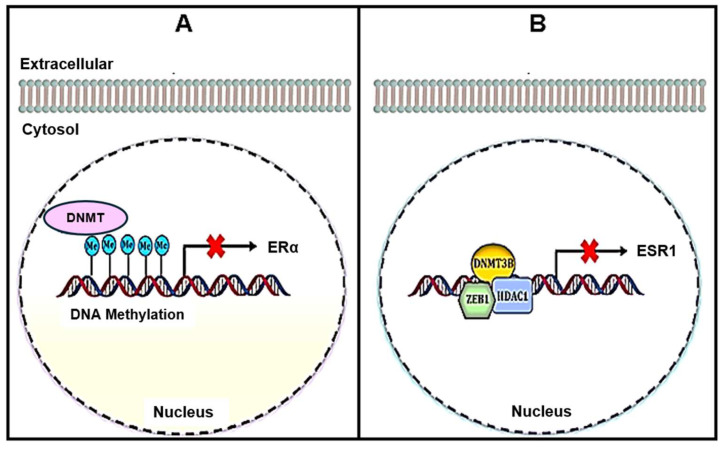
Epigenetic mechanisms control estrogen receptor expression in breast cancer. (**A**) ERα expression inhibition through DNA methylation and (**B**) ESR1 expression inhibition via ZEB1/DNMT 3B/HDAC1 complex. Me, methylated; DNMT, DNA methyltransferase; ERα, estrogen receptor α; ZEB1, zinc finger E-box-binding homeobox 1; ESR1, estrogen receptor 1; HDAC1, histone deacetylase 1; DNMT3B, DNA methyltransferase 3 beta; red (X) in the figure indicates inhibition.

In females, estrogen functions as the principal sex hormone, crucial for reproductive development, bone growth, and metabolic homeostasis. Five main types of estrogen exist: estrone (E1), estrone-sulfate (E1s), 17-β estradiol (E2), estriol (E3), and estetrol (E4) [75]. E1 and E2 are the most prevalent forms of estrogen. E1 mainly serves as an estrogen reserve, capable of bidirectional conversion to the more biologically active E2. E3 and E4 are significantly increased during pregnancy; E3 is usually predominant. Estrogens exert their effects via two primary intracellular estrogen receptors (ERs) encoded by the ESR1 and ESR2 genes: ERα and ERβ, respectively [76]. Approximately 75% of breast cancers are categorized as Erα-positive, generally associated with a more favorable prognosis due to their responsiveness to hormone-based therapies. Conversely, Erα-negative subtypes are linked to less favorable outcomes and more rapid tumor progression [77]. One proposed mechanism contributing to ERα expression suppression is epigenetic silencing through anomalous methylation of the ERα promoter region [78] (Figure 4).

The ESR1 promoter undergoes hypermethylation due to the formation of a complex comprising DNA methyltransferase 3 β (DNMT 3β), zinc finger E-box-binding homeobox 1 (ZEB1), and histone deacetylase 1 (HDAC 1), as shown in Figure 4. Furthermore, increased histone deacetylation results in nucleosome structure condensation, further inhibiting ESR1 transcription [36]. Upon E2 binding, ERα monomers dimerize and translocate to the nucleus, where they function as transcription factors influencing various gene expressions [79]. E2-ERα complexes form dimers that utilize ATP-dependent chromatin remodeling complexes (SWI/SNF) and various histone acetyltransferases (HATs) in estrogen-responsive promoters. These HATs include p300, p160, CREB-binding protein (CBP), and p300/CBP-associated factor (pCAF). This recruitment process ultimately results in enhanced breast cancer cell proliferation and accelerated tumor progression [79]. Early studies have demonstrated that microRNAs (miRNAs) have a significant role in inhibiting estrogen receptor (ER) expression [80,81]. For instance, elevated levels of miR-222 and miR-221 lead to the post-transcriptional inhibition of ERα. Consequently, these miRNAs contribute to the downregulation of various tumor suppressor genes, including tensin homolog (PTEN), phosphatase- and cyclin-dependent kinase inhibitor 1B (CDKN1B), cyclin-dependent kinase inhibitor 1C (CDKN1C), tissue inhibitor of metalloproteinases 3 (TIMP3), BCL2-interacting mediator of cell death (BIM), and forkhead box O3 (FOXO3). Additionally, they promote uncontrolled cell growth that is independent of estrogen [82,83].

### 4.6. Epigenetic Alterations During the EMT

The epithelial–mesenchymal transition (EMT) represents a cellular transformation process through which cells relinquish their epithelial features and attain mesenchymal properties. This process is essential in wound repair, cancer development, and progression. It enhances cancer cell motility, drug resistance, plasticity, and recurrence. Various growth factors (TGF-β, EGF, FGF, IGF, and PDGF) activate signaling cascades that induce EMT transcription factors (SNAI, ZEB, and TWIST), ultimately orchestrating EMT. Research has demonstrated that these transcription factors establish regulatory complexes through interactions with diverse proteins involved in regulating transcriptional processes, involving those that govern epigenetic processes [84]. The interaction between EMT transcription factors and epigenetic regulators plays a critical role in various types of malignancy, including breast cancer, by influencing the expression of EMT-associated genes and inducing EMT [85]. In aggressive breast cancer cells, abnormally elevated methylation levels at CDH1 are observed, which is associated with increased E-cadherin expression [86]. E-cadherin, recognized for its tumor-suppressing properties, enhances cell–cell adhesion between adjacent cells [87]. In breast cancer, G9 suppresses transcription at the E-cadherin promoter through its interaction with Snail [88]. EMT in breast cancer can be reversed by the histone deacetylase inhibitor TSA, which functions by inhibiting Slug expression [89]. Furthermore, BRD4 inhibition results in the suppression of Gli1, an essential component for Snail transcriptional stimulation. This finding recommends that BRD4 influences breast cancer cell aggressiveness via both transcriptional regulation and post-translational mechanisms of Snail [36]. Elucidating the connection between EMT and epigenetics offers novel avenues in developing effective cancer treatments.

## 5. Epigenetics and Cancer Progression

Recent research indicates that a few subsets of cancer cells, identified as cancer stem cells (CSCs), have a crucial role in disease recurrence and metastasis. Breast cancer stem cells (BCSCs), like other CSCs, include a heterogeneous group of malignant cells with substantial proliferative and self-renewal capabilities. Multiple investigations have demonstrated that BCSCs significantly contribute to drug resistance, recurrence, metastasis, and disease initiation [90]. Epigenetic mechanisms are crucial during development for reprogramming stem cells for enabling differentiation into definite cell types and tissues. In certain types of cancer, including breast cancer, abnormal epigenetic modifications commonly enhance the formation of CSCs, which lack the ability to differentiate [91]. Epigenetic processes are critical in regulating the expression of various genes implicated in cancer-related signaling pathways, such as Wnt, Hedgehog, Notch, and Hippo/yes-associated protein 1 (YAP) [92]. Within the breast cancer cells, the promoters of genes associated with Wnt/β-catenin-dependent signaling cascade reactions are frequently hypermethylated [93]. Moreover, a substantial reduction in DNA methylation results in the downregulated expression of inhibitors of Wnt, including Dickkopf-related protein 1 (DKK1), Wnt inhibitory factor 1 (WIF-1), and various frizzled-related proteins (SFRP1-5), in cancer cells. These changes upsurge the activity of Wnt/β-catenin signaling in breast cancer stem cells relative to non-CSCs [94]. These epigenetic modifications significantly impact various signaling pathways related to the tree, involving Wnt and human gonadotropin-releasing hormone (GNRH), thus promoting violence and drug resistance of the cells of breast cancer [95].

## 6. Drug Resistance and Epigenetics

Cancer relapses and poor outcomes are frequently associated with both inherent and acquired resistance to conventional chemotherapy agents. The key factors leading to therapeutic resistance in cancer are postulated to be the emergence of cancer stem cells (CSCs) and the epithelial–mesenchymal transition (EMT) process. Accumulating evidence indicates that epigenetic dysregulation also has a significant role in drug resistance for various cancers, including breast cancer [20]. In breast tumors, the EZH2-mediated repression of estrogen receptor 1 (GREB1), an ERα cofactor, results in tamoxifen resistance [96]. The loss of translocation 11-11 2 (TET2) in breast cancer cells leads to reduced ERα expression, consequently causing endocrine resistance [78]. Histone acetyltransferase lysine acetyltransferase 2A (LAT2A) enhances tamoxifen drug resistance during the treatment course of breast cancer by decreasing p53 stability and increasing breast cancer 1 (AIB1) expression [36]. The LSD1 demethylase is involved in regulating breast CSC self-renewal, thereby contributing to the development of chemoresistance [97]. Furthermore, in breast cancer, lysine-specific demethylase 1A (LSD1) modulates EMT through its interaction with protein kinase Cθ (PKC-θ), thus promoting drug resistance [98].

## 7. Progress in Epigenetic Therapy

**Figure 5:**
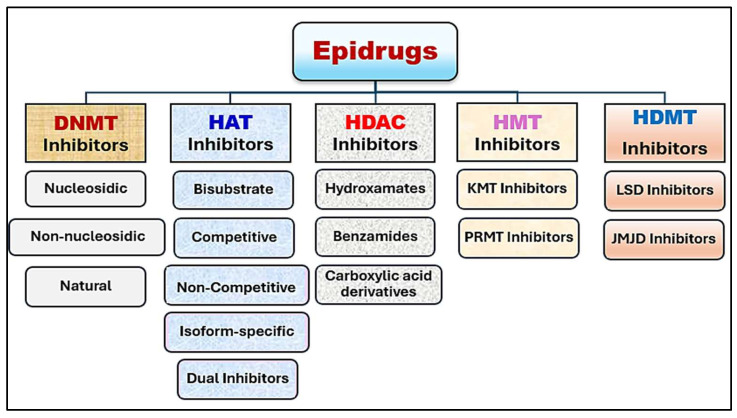
Epidrugs available for treatment of breast cancer categorization.

Epigenetic therapy is an emerging medical field that utilizes chemical compounds to rectify epigenetic alterations through epigenetic modulators. These medications offer precise targeting capabilities and create new avenues for individualized treatment [99]. They can influence genetic activity by addressing abnormal gene expression linked to cancer, neurodegenerative disorders, and autoimmune conditions [100]. Epidrugs are categorized into five main types: DNA methyltransferase (DNMT) inhibitors, histone deacetylase (HDAC) inhibitors, histone acetyltransferase (HAT) inhibitors, histone demethylase (HDM) inhibitors, and histone methyltransferase (HMT) inhibitors [95] (Figure 5).

Initial epidrugs exhibited limitations such as suboptimal pharmacokinetic properties, specificity, and bioavailability. This resulted in the development of enhanced epidrugs with extended half-lives, reduced cytotoxicity, diminished adverse effects, and enhanced inhibitory capabilities [101]. The most recent generation of epidrugs targets epigenetic factors categorized as writers, readers, and erasers. Erasers function by removing methyl or acetyl groups that writers have added to DNA or histones, while readers identify and regulate binding interactions [101]. Table 1 provides a list of epidrugs used in breast cancer treatment.

### 7.1. DNA-Modifying Drugs

The process of DNA methylation involves the attachment of a methyl group to the fifth carbon of cytosine, a reaction catalyzed by DNA methyltransferases (DNMTs), which include DNMT1, DNMT3A, DNMT3B, and DNMT3L. This modification inhibits gene expression by impeding the binding of transcription factors to DNA [104]. DNMT inhibitors function by inserting themselves between DNA base pairs, thereby preventing the methylation of CpG islands in promoter sequences. Decitabine and azacitidine are examples of first-generation DNMT inhibitors that utilize this mechanism [105]. DNMT inhibitors are classified into three categories: non-nucleoside, nucleoside, and natural compounds. Certain natural substances, such as epigallocatechin-3-gallate (EGCG), catechins, and quercetin, have demonstrated the ability to inhibit DNMT1. This inhibition results in DNA demethylation, reactivation of tumor suppressor genes, and a reduction in breast cancer cell proliferation [106]. Hydralazine, a traditional non-nucleoside compound used for hypertension treatment, also reduces DNMT expression in mammals [107]. When combined with valproic acid, a mild demethylating agent, hydralazine diminishes cancer cell survival in a dose-dependent manner [108]. The nucleoside DNMT inhibitor zebularine enhances p21 expression, reduces cyclin-D expression, induces apoptosis, and arrests MCF-7 and MDA-MB 231 cells in the S phase [109]. The antiarrhythmic agent procainamide, a non-nucleoside DNMT analog, increases the responsiveness of Eα-positive breast cancer cells to the estrogen receptor antagonist, tamoxifen, by increasing ERβ expression [110]. Olsalazine, utilized in the treatment of ulcerative colitis, elevates CDH1 gene expression, which encodes E-cadherin, thus inhibiting EMT in MDA MB-231 breast cancer cells [111]. Liraglutide, the common antidiabetic drug, acts as an inhibitor for DNMT by attenuating methylation in the promoter regions of ADAM33, CDH1, and ESR1 in MDA-MB-231, MCF-7, and MDA-MB-436 cell lines [112]. Furthermore, in silico research has identified five potential DNMT1 inhibitors: C-2140, C-1723, C-5769, C-2129, and C 7756 [113].

### 7.2. HAT and HDAC Inhibitors

Histone acetyltransferase (HAT) and histone deacetylase (HDAC) are important enzymes that play a role in gene expression regulation and chromatin restructuring [114]. HATs facilitate adding acetyl groups from acetyl-CoA to lysine residues. This process leads to DNA sequence transcriptional activation due to a loosened structure of chromatin [114]. Conversely, HDACs remove acetyl groups from histones, resulting in a more compact and stable structure of chromatin, which suppresses transcription [115]. Disruptions in the equilibrium between these enzymes can result in severe pathological conditions, including cancer [101]. Various naturally occurring inhibitors of HAT, such as garcinol, curcumin, anacardic acid, and carnosol, are currently under investigation for their potential therapeutic applications in breast cancer treatment. Carnosol, a polyphenolic compound found in high concentrations in sage, oregano, and rosemary, is hypothesized to inhibit p300 by obstructing its acetyl CoA binding site, consequently inducing histone hypoacetylation in breast cancer cell lines [116]. Garcinol has been demonstrated to effectively inhibit CBP/p300-mediated p53 acetylation in MCF-7 breast cancer cell lines. Curcumin also inhibits p300/CBP activity, arresting MCF-7 breast cancer cells in the G2/M phase [117]. HDAC inhibitors are categorized into three classes: hydroxyamates (e.g., panobinostat, belinostat, vorinostat, CUDC-10, dacinostat, tefinostat, and quisinostat), benzamides (e.g., chidamide, mocetinostat, entinostat, and tacedinaline), and carboxylic-acid-based inhibitors (e.g., Phenylbutyric acid, valproic acid, and Pivanex) [118]. Vorinostat is a prominent first-generation HDAC inhibitor that targets class I and II HDAC families and needs a Zn2+ ion for its substrate catalytic binding. This substance reduces ERα expression by targeting it for degradation via ubiquitin–proteasome signaling, resulting in decreased cell proliferation, migration, and invasion [119]. Quisinostat, a next-generation inhibitor of class I and II HDACs, inhibits cancer stem cell self-renewal by enhancing histone H1 production without any hazard to the normal stem cells [120]. Another class I and II HDAC inhibitor, Tacedinaline, demonstrates potential in downregulating the BIRC5 gene, which is frequently overexpressed in resistant breast cancer cells, and in suppressing apoptotic protein expression [121]. When utilized together with capecitabine, Mocetinostat, an inhibitor of HDAC I and IV, has demonstrated the capacity to induce apoptosis in breast cancer cell lines [122]. Chidamide, a benzamide class HDAC inhibitor targeting HDAC 1, 2, 3, and 10, has exhibited promising efficacy in treating HER+ breast cancer patients when used in conjunction with exemestane [123].

### 7.3. HMT and HDMT Inhibitors

Histone methyltransferases (HMTs) have an important role in adding methyl groups to specific amino acid residues on proteins of histones. Examples of HMT are lysine methyltransferases (KMTs) and arginine methyltransferases (RMTs), which use S-adenosyl l-methionine (SAM) as a methyl donor. At present, GSK3326595, a PRMT5 inhibitor, is under clinical trials for the treatment of early HR+ cancer. Furthermore, JNJ-64619178, another potent PRMT5 inhibitor, is being evaluated in clinical trials for advanced tumors [124]. Pemrametostat, an experimental oral small molecule inhibitor, targets PRMT5 by inhibiting arginine methylation in histones H4, H3, and H2A, thereby inhibiting tumor cell growth. Sinefungin, a natural EHMT1/2 inhibitor, is synthesized by Streptomyces incamatus and S. griseolus [36]. Various inhibitors primarily target EZH1/2 activity, including DS-3201b, ORS1/ORS2, and UNC1999, whereas other compounds such as GNA022, ANCR, FBW7, and ZRANB1 focus on EZH2 degradation [125]. Histone demethylases (HDMTs) are classified into two groups depending on their specificities: flavinadenine-dependent demethylases (FADs) and iron–oxygen-dependent 2-oxoglutarate demethylases, which include the Chumon domain [126]. Phenelzine, an MAO inhibitor antidepressant, irreversibly suppresses LSD1 activity and reduces mesenchymal markers when used in combination with nab-paclitaxel for treating metastatic breast cancer patients. Studies have demonstrated that it also suppresses cancer stem cells and decreases migrating tumor cells responsible for metastasis [127]. Pargyline, another monoamine oxidase inhibitor, inhibits LSD1 activity, leading to a concentration-dependent decrease in cellular development and increased expression of apoptotic proteins [128]. Additionally, it inhibits cell multiplication, growth, and invasive behavior [129]. ORY-1001 (iadademstat), an LSD1 inhibitor, reduces the expression of androgen receptors in breast cancer cell lines like BT549 and MDA-MB-231. Moreover, it decreases cell proliferation and promotes apoptotic processes [130].

### 7.4. Epidrug Combination Therapy in Breast Cancer

The integration of epidrugs with chemotherapy or immunotherapy demonstrates potential in addressing drug resistance and enhancing overall treatment efficacy [131]. This combined approach holds significant promise in breast cancer treatment, facilitating the development of personalized and efficient therapeutic strategies. Multiple combinations are currently undergoing clinical trials to establish optimal dosages and evaluate long-term safety and efficacy (Table 2). 

Studies have demonstrated that combinations of HDAC inhibitors exhibit greater efficacy than when utilized individually [145]. Concurrent treatment with the estrogen receptor C29 α (ERRα) inhibitor with a DNMT inhibitor such as 5-aza-2′-deoxycytidine (Decitabine) prevents cross-linking between DNMT1 and ERRα, promoting ERRα stability, which subsequently leads to DNA methylation. The interaction of these compounds facilitates the reactivation of the tumor suppressor gene Interferon Regulatory-4 (IRF4), which inhibits breast cancer cell proliferation [144]. Researchers are currently investigating the efficacy of panobinostat, a histone deacetylase (HDAC) inhibitor, in conjunction with trastuzumab for treating HER-positive metastatic breast cancer through clinical trials [146]. Two additional compounds, vorinostat and valproic acid, have demonstrated the capacity to enhance trastuzumab’s ability to induce antibody-dependent phagocytosis and cell-mediated cytotoxicity. Furthermore, these compounds have been observed to decrease the expression of MCL1, an anti-apoptotic protein involved in the differentiation of myeloid leukemia cells, and in HER-positive SKBR3 cells [133]. Research indicates that combining entinostat with lapatinib can reduce colony-forming ability by inhibiting the FOXO3-mediated expression of phosphorylated AKT and Bim, thus promoting apoptosis [139]. When used in combination, vorinostat functions as an HDAC inhibitor while olaparib targets polyadenosine 5′ diphosphoribose polymerase (PARP). This combination disrupts cancer cells’ DNA repair mechanisms, leading to tumor cell death [119]. Studies have demonstrated that valproic acid, a known HDAC inhibitor, enhances capecitabine’s anticancer effects by increasing thymidine phosphorylase (TP) expression. TP plays a vital role in the conversion of 5′-DFUR into 5 fluoro-2-deoxyuridine monophosphate (FdUMP) that hinders thymidine production by disrupting DNA replication through the inhibition of thymidylate synthase (TS). The combined effect of these two drugs leads to apoptosis and damage to RNA and DNA in several breast cancer cell lines [132]. A phase 2 clinical study demonstrated that azacitidine and entinostat work together to suppress DNA synthesis and class I HDAC activity [142]. Research also indicated that panobinostat, another HDAC inhibitor, enhances trastuzumab’s efficacy by triggering a CXCR3-mediated NK cell and IFNγ-dependent immune response in breast cancer [120]. In TNBC cells, the combination of entinostat and atezolizumab targets both HDAC activity and PD-L1 [140]. Furthermore, when combined with panobinostat, letrozole, a well-established aromatase inhibitor, effectively restored the expression of ERα and an enhanced responsiveness to endocrine treatment was observed. This phenomenon was characterized by the increased acetylation of histones H4 and H3, leading to the depression of progression in tumor cells lacking ER expression [141]. Studies have shown that vorinostat enhances paclitaxel’s antiapoptotic effects through histone and α-tubulin acetylation. Additionally, it impedes Hsp90’s interactions with HER2, c-RAF, and AKT via Hsp90’s proteasomal deprivation, while also exhibiting antiangiogenic properties by influencing vascular endothelial growth factor (VEGF) cascade signaling [135]. Research has demonstrated that the combination of diverse therapeutic agents can enhance the efficacy of breast cancer treatments. For instance, in patients with advanced localized breast cancer receiving doxorubicin and cyclophosphamide therapy, the addition of hydralazine (a DNA demethylating agent) and magnesium valproate (an HDAC inhibitor) functioned synergistically to activate genes and reduce overall C5 methylation [137]. A novel therapeutic approach utilizing a combination of romidepsin (a histone deacetylase inhibitor), gemcitabine (an agent that inhibits DNA synthesis), and cisplatin (a compound that damages DNA) was demonstrated to induce apoptosis in TNBC cell lines. This effect was mediated through the excessive production of reactive oxygen species (ROS) and PARP proteolytic cleavage [147]. Furthermore, investigations have indicated that utilizing the DNMT inhibitor decitabine in conjunction with doxorubicin effectively reduced DNMT1 activity, DNA methylation, and cancer cell proliferation [134].

### 7.5. RNA-Based Therapies

A novel approach to gene activation involves the utilization of non-coding RNAs to specifically enhance gene expression. Research has demonstrated that certain tumor suppressor genes are frequently silenced in breast cancer through promoter hypermethylation, rendering the restoration of gene expression a potentially significant advancement in epigenetic cancer therapy [148]. Initial studies employing vectors encoding RNA-polymerase-II-dependent long antisense non-coding RNAs that complement promoter sequences have demonstrated the capacity to reverse DNA methylation and reactivate gene expression [149]. When mice with neoplasms were preinjected with these vectors, tumor growth was suppressed and survival rates increased. Although still in its early stages, this method exhibits promise for preventing breast cancer by targeting premalignant mammary lesions [150].

Double-stranded RNA initiates a process known as RNA interference, which functions to suppress gene expression, which inhibits gene expression at the post-transcriptional level [151]. The two types of RNA involved in this process are microRNAs (miRNAs) and small-interfering RNAs (siRNAs), both approximately 20–25 nt in length. Studies have revealed that miRNA expression levels can predict clinical outcomes in breast cancer. This observation led to the hypothesis that manipulating miRNA levels—either by increasing certain miRNAs to regulate abnormal gene expression or decreasing oncogenic miRNAs—could serve as a targeted therapy for breast cancer [152]. However, a comprehensive understanding of miRNAs in breast cancer is necessary prior to implementing any miRNA-based therapy, thus limiting its current potential as a promising epigenetic treatment for breast cancer [153]. As examples of RNA-based therapies, the Food and Drug Administration (FDA) approved four RNAi-based drugs: patisiran, givosiran, lumasiran, and inclisiran. Each of these siRNA drugs targets a specific mRNA transcript to address a particular disease [154]. Moreover, multiple siRNA agents, including fitusiran, nedosiran, teprasiran, tivanisiran, and vutrisiran, have advanced to phase III clinical trials. Numerous other RNAi-based therapeutics are progressing through early-stage clinical trials or preclinical development. Notwithstanding these advancements, the primary challenge impeding clinical success has consistently been the effective delivery of RNAi therapeutics [154].

## 8. Obstacles and Constraints

### 8.1. Insufficient Selectivity

At present, all medications that inhibit epigenetic machinery enzymes lack target-specific action, particularly HDAC inhibitors. Research has demonstrated that a specific HDAC inhibitor only modifies the acetylation state of a small portion of expressed genes, with the majority of transcriptional effects being indirect. This non-specificity raises concerns, as alterations in histone and/or non-histone protein acetylation may disrupt gene expression in ways that could adversely affect unintended pathways. Furthermore, acetylated p53 is known to activate transcription for several genes, including p21 [155]. Given that p53 mutation or inactivation is prevalent in many cancers, the effects of HDAC inhibitors on p53-independent pathways must be considered. A more comprehensive understanding of individual HDAC enzyme roles and the development of enzyme-specific inhibitors would facilitate the determination of the viability of HDAC inhibitors as anticancer treatments [156]. While specificity is less problematic for DNMT inhibitors, global DNA demethylation is potentially hazardous, and the extent to which it can be safely induced and its consequences remain undetermined [157].

### 8.2. Resistance Emergence

Studies on tumor resistance mechanisms to epigenetic drugs are still in their nascent stages. Some tumors are known to resist HDAC-inhibitor-induced apoptosis. One potential mechanism involves the inactivation of the p21 tumor suppressor following treatment with DNA methyltransferase inhibitors in tumors with p21 gene mutations. Given that DNMT inhibitors cause gene demethylation and re-expression, this or similar effects on other genes could pose a significant treatment challenge [158]. Another study revealed that increased H2AX histone variant phosphorylation can lead to HDAC inhibitor resistance [159]. Moreover, as discussed in Section 6, HDAC inhibitors may interfere with DNA repair processes following chemotherapy or radiation therapy. If DNA repair machinery is confirmed as a target of HDAC inhibitors in tumor cells, it can potentially result in the development of HDAC inhibitors as adjuvant therapy. However, the feasibility and safety of this approach remain uncertain, and it may negatively impact healthy cells damaged during these treatment [160].

### 8.3. High Cost

One of the significant challenges limiting the widespread clinical application of epidrugs is their high cost, which presents a substantial barrier to accessibility and affordability for numerous patients. The development and production of epidrugs, such as DNA methyltransferase inhibitors (e.g., azacitidine) and histone deacetylase inhibitors (e.g., vorinostat), involve complex manufacturing processes and rigorous clinical trials, contributing to their elevated prices [161]. Furthermore, the personalized nature of epigenetic therapies often necessitates tailored treatment regimens, further increasing expenses. For patients in low- and middle-income countries, as well as those with limited insurance coverage, the high cost of these therapies can be prohibitive, restricting their use to a small subset of individuals. This economic burden underscores the need for cost-effective strategies, such as generic drug development, government subsidies, or innovative pricing models, to ensure equitable access to these promising treatments. Addressing the financial challenges associated with epidrugs is crucial for maximizing their clinical potential and improving outcomes for a broader population of cancer patients [161].

## 9. Future Directions and Opportunities

### 9.1. Personalized Epigenetic Therapies

The ongoing accumulation of data and advancements in techniques for examining the cancer epigenome are anticipated to be utilized in a novel era of individualized cancer treatment based on molecular information. As the ability to forecast treatment responses using molecular tumor profiles improves, the results of efforts to identify both genetic and epigenetic characteristics specific to various cancers are becoming evident. It is expected that numerous predictive models will be developed, incorporating information on both genetic and epigenetic features of a given cancer as well as data on the nature of the epigenetic abnormality involved [162]. These models will offer a degree of accuracy in predicting whether a tumor with specific molecular traits will respond to epigenetic therapy using a single agent, potentially aiding in patient selection for single-agent therapy versus more toxic and less specific combination treatments [163]. The current low success rate of breast cancer therapies is primarily attributed to their design for the average patient, disregarding the various breast cancer subtypes known to have differing prognoses and treatment responses. The effectiveness of epigenetics will be evaluated by the expression of genes known to be inappropriately silenced by DNA promoter hypermethylation and/or histone hypoacetylation (e.g., ER, pS2, hTERT, cyclin D2), and the reactivation of these genes will be linked to changes in DNA methylation and histone acetylation at target promoters [164]. This information can be utilized to create personalized epigenetic therapy programs aimed at achieving the optimal re-expression outcome for each patient. The potential development of individual epigenetic profiles to predict tumor aggressiveness and the need for specific epigenetic therapy will enable the further customization of epigenetic treatments for optimal patient outcomes [165]. The future of breast cancer treatment lies in personalized therapies, including epigenetic approaches. By targeting individual epigenetic modifications in breast cancer cells, it may be possible to design therapies that specifically address the primary abnormality of a patient’s tumor, enhancing effectiveness and minimizing side-effects [166]. This can be achieved through advancements in epigenome editing technologies currently under development, such as zinc finger nucleases and synthetic transcription factors designed to target specific DNA sequences or histone modifications. Additionally, it is feasible to design personalized combination therapies using epigenetic and genetic targets to restore repressive chromatin states at key gene loci in cancer pathways, selectively eliminating cancer cells. One example is the simultaneous targeting of ER+ breast cancer cells with HDAC inhibitors to enhance ER expression, thereby increasing the tumor’s responsiveness to hormonal therapy [167]. Future studies such as these may offer a wide range of different epigenetic therapies specific to patients with different subtypes of breast cancer. An international initiative, the Human Epigenome Project, is currently supporting much research in this area and aims to generate high-resolution maps of human epigenomic changes at a genome-wide scale. As technology continues to develop, it will become increasingly possible to assess epigenetic changes in patients’ tumors. This, in turn, will pave the way for predictive epigenetic therapy programs with personalized patient therapy designed to reverse epigenetic changes at specific genetic loci across the genome [168]. Furthermore, nutraceuticals, which are food-derived bioactive compounds with potential health benefits, have emerged as promising candidates for epigenetic therapy in breast cancer. These compounds can modulate epigenetic mechanisms such as DNA methylation, histone modifications, and non-coding RNA expression, potentially influencing gene expression patterns associated with cancer development and progression. Several nutraceuticals—including polyphenols (e.g., resveratrol, kaempferol, curcumin, quercetin), isothiocyanates (e.g., sulforaphane), and other phytochemicals—have demonstrated the capacity to target epigenetic enzymes and alter epigenetic marks in breast cancer cells [169]. By reversing aberrant epigenetic modifications, these compounds may facilitate the restoration of normal gene expression, suppress oncogenes, reactivate tumor suppressor genes, and modulate signaling pathways involved in cell proliferation, apoptosis, and metastasis. The potential advantages of nutraceuticals in epigenetic therapy include their natural origin, relatively low toxicity, and ability to target multiple epigenetic mechanisms simultaneously [169]. However, further research is necessary to fully elucidate the mechanisms of action, optimize dosing regimens, and evaluate the long-term efficacy and safety of nutraceuticals as epigenetic modulators in breast cancer prevention and treatment.

### 9.2. Combination Therapies

Epigenetic therapies, owing to their relatively low toxicity, are well suited for use in conjunction with conventional cytotoxic chemotherapy. Research has demonstrated that when utilized in combination, epigenetic therapy can enhance the efficacy of chemotherapy. A significant synergistic effect has been observed between DNMT inhibitors and histone deacetylase inhibitors, leading to enhanced growth inhibition and apoptosis in several types of cancerous cells. The combined application of these drug classes produces a more substantial impact than either alone, suggesting that this dual approach could be highly effective across a broad spectrum of cancers [170]. This synergy provided the foundation for a phase I trial combining azacitidine and entinostat in the treatment of advanced leukemias, MDS, and CMML. Similar enhancements have been noted with other epigenetic drug classes, including histone deacetylase inhibitors and retinoid therapy, indicating that all epigenetic therapy types could potentially complement chemotherapy [171]. In cancer treatment, combination therapies are frequently employed to target tumors from multiple angles or to mitigate the likelihood of drug-resistant tumor cells emerging [172]. Conventional cytotoxic therapy has often been combined with novel molecularly targeted agents in an effort to improve efficacy. While the outcomes of these combinations vary, there is substantial support for utilizing combinations of epigenetic therapies [173].

### 9.3. Targeting Epigenetic Plasticity

Plasticity describes a system’s capacity to adapt to environmental challenges by transitioning between distinct functional states. The observation that human tumors display a specific DNA methylation phenotype, which can be maintained in culture or in immunocompromised mice, has generated considerable interest in the possibility of reprogramming abnormal DNA methylation in cancer cells [174]. Achieving this will necessitate a comprehensive understanding of the signal transduction pathways that regulate DNA methyltransferase and histone-modifying enzyme activity, as well as the ability to manipulate these pathways using pharmacological agents. An alternative and more comprehensive strategy would be to focus on the proteins that interpret the methylation code. Elevated levels of DNA methylation have been accompanied with the silencing of the tumor suppressor gene [38].

## 10. Conclusions

Breast cancer is a complex disease frequently characterized as debilitating, refractory to treatment, unresponsive to conventional therapies, and prone to recurrence. Over the past decade, extensive research into the impact of epigenetic dysregulation on various forms of cancer has elucidated the substantial clinical potential of epidrugs. Epigenetic regulators—such as DNA methylation, non-coding RNAs, and histone modification—play a pivotal role in the development of breast cancer. Considering the intricate interplay between epigenetic regulators and breast cancer progression and proliferation, EMT-TFs and targeting epigenetic enzymes offer a viable and efficacious approach to reversing the EMT process. However, the utilization of epidrugs as monotherapy for solid tumors, including breast cancer, frequently presents considerable challenges because of their limited effectiveness, poor tolerability, and possible adverse and toxic effects. Consequently, a more systematic approach to combining epidrugs with other cancer treatments is necessary. Epigenetic therapy demonstrates significant potential in improving breast cancer prognosis by specifically targeting certain epigenetic alterations, restoring normal gene expression patterns, and increasing the vulnerability of tumor cells to traditional treatments. Further basic molecular research and clinical trials are required to elucidate the comprehensive role of epigenetic treatment in developing personalized treatment strategies for breast cancer patients.

## Figures and Tables

**Table 1:** Available epidrugs for breast cancer therapy.

Epidrug Generation	Class	Compounds	Reference
First generation	DNMT inhibitors	Azacitidine, decitabine	[101,102]
	HDAC inhibitors	Trapoxin A, trichostatin, vorinostat, romidepsin	[101,102]
Second generation	DNMT inhibitors	Hydralazine, guadecitabine, CP-4200, zebularine	[102,103]
	HDAC inhibitors	Panobinostat, belinostat, dacinostat, quisinostat, CUDC-101, entinostat, chidamide, tacedinaline, tefinostat, pivanex, butyric acid, valproic acid, phenylbutyric acid	[102,103]
Third generation	KDM inhibitors	Clorgyline, GSK2816126, bizine, KDM5-C70, ORY-101, JIB-04, 4SC-202, tranylcypromine, pargyline	[102,103]
	KMT inhibitors	Tazemostat, Pinometostat, Sinefungine, BIX-01294, GSK2816126, GSK3326595, GSK3368715, JNJ64619178, DZNep, CPI360, GSK343, EPZ004777, UNC0638, UNC0224	[102,103]
	Bromodomain ligands	CPI-0610, RVX-280, I-BET762, OTX015	[102,103]

HDAC: histone deacetylase; DNMT: DNA methyltransferase; KMT: lysine methyltransferase; KDM: lysine demethylase; I-BET: inhibitor of BET (bromodomain and extraterminal) proteins; DZNep: 3-deazaneplanocin A; RVX-280, resverlogix-280; CP-4200, GSK2816126, ORY-101, 4SC-202, JIB-04, KDM5-C70, GSK2816126, GSK3326595, GSK3368715, JNJ64619178, DZNep, CPI360, GSK343, BIX-01294, EPZ004777, UNC0224, and UNC0638 are names of drugs or investigational compounds utilized as epidrugs.

**Table 2:** Epidrugs utilized in conjunction with various chemotherapeutic agents for breast cancer treatment.

S. No.	Drug Combination	Mechanism of Combined Action	Reference
1	Decitabine + zebularine	Disturbance of colony formation potential and cell proliferation	[109]
2	Exemestane + entinostat	Aromatase inhibitor and HDAC class I inhibitor	[101]
3	Zebularine + vorinostat	Disturbance of colony formation potential and cell proliferation	[109]
4	Capecitabine + valproic acid	Increase in TP, decrease in TS enzymes, decrease in thymidine synthesis	[132]
5	Valproic acid + vorinostat + Trustuzumab	Decrease in MCL1, increase in ADCC and ADCP	[133]
6	Paclitaxel + liraglutide	Stimulation of cellular demethylation via the abolition of DNMTs and transcription of ADAM33, CDH1, and ESR1 genes, leading to inhibition of cell migration and viability	[112]
7	Methotrexate + liraglutide	Stimulation of cellular demethylation via the abolition of DNMTs and transcription of ADAM33, CDH1, and ESR1 genes, leading to inhibition of cell migration and viability	[112]
8	Vorinostat + olaparib	PARP, HDAC	[119]
9	UNC0638 + tacedinaline	Modulation of G9a and class I HDAC, inhibition of BIRC5, and stimulation of GADD45A	[121]
10	Doxorubicin + decitabine	Inhibition of tumor proliferation, DNMT1 activity, and DNA methylation	[134]
11	Paclitaxel + vorinostat	Activation of acetylation of both α-tubulin and histone, proteasomal breakdown of Hsp90, enhancement in antiangiogenetic effect	[135]
12	Nab-paclitaxel + phenelzine	Inhibition of CSC generation by downregulation of mesenchymal markers	[127]
13	Gemcitabine + romidepsin + cisplatin	TNBC cell apoptosis via ROS generation	[136]
14	Doxorubicin + hydralazine + cyclophosphamide + magnesium valproate	DNA demethylation and inhibition of HDAC activity, decrease in C5me content	[137]
15	Panobinostat + trustuzumab	Stimulation of NK-cell-mediated immune response	[138]
16	Lapatinib + entinostat	Transcriptional activation of FOXO3 and Bim	[139]
17	Atezolizumab + entinostat	Downregulation of HDAC activity and PD	[140]
18	Panobinostat + letrozole	BC cell sensitization to hormonal therapy, stimulation of H3, H4 acetylation, inhibition of aromatase activity	[141]
19	Entinostat + azacitidine	Inhibition of DNA synthesis and HDAC class I activity	[142]
20	Chidamide + mocetinostat	Inhibition of aromatase and HDAC subtype activities	[143]
21	Decitabine + C29	Inhibition of DNMT1 and ERRα	[144]
22	Mocetinostat + capecitabine	Stimulation of cell apoptosis pathway by inhibiting HDAC1, BC12, Akt, c-myc, and PI3K and stimulating C-Parp, cas-7, Bax, Cas-9, Pten, Cas-3, and p-53	

HDAC: histone deacetylase; TP: thymidine phosphorylase; TS: thymidylate synthase; MCL1: myeloid cell leukemia 1; ADCP: antibody-dependent cellular phagocytosis; ADCC: antibody-dependent cellular cytotoxicity; DNMT: DNA methyltransferase; BIRC5: Baculoviral IAP repeat-containing 5; GADD45A: growth arrest and DNA-damage-inducible alpha; ROS: reactive oxygen species; CSC: cancer stem cell; PARP: poly ADP-ribose polymerase; TNBC: triple-negative breast cancer; NK: natural killer; FOXO3: forkhead box O3; Bim: BCL2-interacting mediator; PD: programmed death; DNMT1: DNA methyltransferase 1; ERRα: estrogen receptor-α; Akt: protein kinase B; PI3K: phosphoinositides 3-kinase; BCL2: B-cell lymphoma 2; UNC0638: a specific inhibitor of G9a and GLP.

## Data Availability

No new data were created.
